# The Dynamics of β-Amyloid Proteoforms Accumulation in the Brain of a 5xFAD Mouse Model of Alzheimer’s Disease

**DOI:** 10.3390/ijms23010027

**Published:** 2021-12-21

**Authors:** Anna E. Bugrova, Polina A. Strelnikova, Maria I. Indeykina, Alexey S. Kononikhin, Natalia V. Zakharova, Alexander G. Brzhozovskiy, Evgeny P. Barykin, Stanislav I. Pekov, Maria S. Gavrish, Alexey A. Babaev, Anna M. Kosyreva, Anna Y. Morozova, Daniil A. Degterev, Vladimir A. Mitkevich, Igor A. Popov, Alexander A. Makarov, Evgeny N. Nikolaev

**Affiliations:** 1 Emanuel Institute of Biochemical Physics, Russian Academy of Science, 119334 Moscow, Russia; anna.bugrova@gmail.com (A.E.B.); pauline.strel@gmail.com (P.A.S.); mariind@yandex.ru (M.I.I.); nvzakharova@yandex.ru (N.V.Z.); 2 Moscow Institute of Physics and Technology, 141701 Dolgoprudny, Russia; stanislav.pekov@forwe.ru (S.I.P.); hexapole@gmail.com (I.A.P.); 3 Engelhardt Institute of Molecular Biology, Russian Academy of Science, 119991 Moscow, Russia; a.kononikhin@skoltech.ru (A.S.K.); eugbar96@gmail.com (E.P.B.); daniil_degterev@mail.ru (D.A.D.); mitkevich@gmail.com (V.A.M.); 4 Skolkovo Institute of Science and Technology, 121205 Moscow, Russia; agb.imbp@gmail.com; 5 Institute of Neuroscience, Lobachevsky State University of Nizhny Novgorod, 603022 Nizhny Novgorod, Russia; mary_gavrish@mail.ru (M.S.G.); alexisbabaev@list.ru (A.A.B.); 6 Research Institute of Human Morphology, 117418 Moscow, Russia; kosyreva.a@list.ru; 7 Department of Basic and Applied Neurobiology, Federal Medical Research Center for Psychiatry and Narcology, 119034 Moscow, Russia; hakurate77@gmail.com; 8 Mental-Health Clinic No. 1 Named after N.A. Alexeev of Moscow Healthcare Department, 117152 Moscow, Russia; 9 Neurological Department, Loginov Moscow Clinical Scientific Center, 111123 Moscow, Russia; 10 V.L. Talrose Institute for Energy Problems of Chemical Physics, N.N. Semenov Federal Center of Chemical Physic, Russian Academy of Sciences, 119334 Moscow, Russia

**Keywords:** Alzheimer’s disease, proteomics, mouse brain, β-amyloid, senile plaques, proteoforms, isoforms, isoD7, post-translational modifications (PTM), mass spectrometry

## Abstract

Alzheimer’s disease (AD) is the leading cause of dementia among the elderly. Neuropathologically, AD is characterized by the deposition of a 39- to 42-amino acid long β-amyloid (Aβ) peptide in the form of senile plaques. Several post-translational modifications (PTMs) in the N-terminal domain have been shown to increase the aggregation and cytotoxicity of Aβ, and specific Aβ proteoforms (e.g., Aβ with isomerized D7 (isoD7-Aβ)) are abundant in the senile plaques of AD patients. Animal models are indispensable tools for the study of disease pathogenesis, as well as preclinical testing. In the presented work, the accumulation dynamics of Aβ proteoforms in the brain of one of the most widely used amyloid-based mouse models (the 5xFAD line) was monitored. Mass spectrometry (MS) approaches, based on ion mobility separation and the characteristic fragment ion formation, were applied. The results indicated a gradual increase in the Aβ fraction of isoD7-Aβ, starting from approximately 8% at 7 months to approximately 30% by 23 months of age. Other specific PTMs, in particular, pyroglutamylation, deamidation, and oxidation, as well as phosphorylation, were also monitored. The results for mice of different ages demonstrated that the accumulation of Aβ proteoforms correlate with the formation of Aβ deposits. Although the mouse model cannot be a complete analogue of the processes occurring in the human brain in AD, and several of the observed parameters differ significantly from human values supposedly due to the limited lifespan of the model animals, this dynamic study provides evidence on at least one of the possible mechanisms that can trigger amyloidosis in AD, i.e., the hypothesis on the relationship between the accumulation of isoD7-Aβ and the progression of AD-like pathology.

## 1. Introduction

Alzheimer’s disease is the most socially significant neurodegenerative disease of the elderly [1], with about 50 million patients worldwide. Its molecular pathogenesis is characteristic of proteinopathies, and it is closely related to the dyshomeostasis and aggregation of the beta-amyloid peptides (Aβ) and tau proteins in the brain [2]. For about 30 years, the formation of Aβ oligomers and plaques was considered the key pathological change that promoted neurotoxicity and induced the further steps of the pathogenic cascade, which involves tau hyperphosphorylation and neuroinflammation [3,4,5,6,7]. However, many studies on the pathogenesis and molecular aspects of amyloid plaque formation are still ongoing and are dictated by the high need to find effective therapeutic agents that could contribute to the destruction of toxic amyloid aggregates and/or prevent their formation [7,8,9].

A number of Aβ PTMs that enhance the assembly of Aβ oligomers are considered potential triggers of AD pathology [10]. They include almost all PTMs in the N-terminal region, such as the isomerization and racemization of D1 and D7, the pyroglutamylation of E3 and E11, the phosphorylation and racemization of S8, the nitration of Y10, and the formation of dityrosine [10,11].

The deamidation of Asn residues is a remarkable and prevalent phenomenon that occurs during protein aging. Considering the metal-binding ability of Asn residues, deamidation can be involved in the pathogenesis of AD [12].

The oxidation of Aβ at Met35 also plays an important role in AD oxidative stress events and is associated with neurotoxicity. Since Met35 is involved in free radical production, its substitution with Val or Leu residues precludes free radical production, oxidative stress, and toxicity of Aβ [13]. On the other hand, AβMet35-sulfoxide has been shown to impede fibril formation [14]. Presumably, oxidized Met35 is more frequent in monomers and dimers than in larger fibrillary structures [15].

Iso-aspartates and pyroglutamates are the most abundant Aβ PTMs [16,17], which have been studied by a number of immunohistochemical and MS-based approaches [10,11,17,18,19,20,21,22]. Since most PTMs are located in the N-terminal zinc-binding domain, the use of Aβ (1–16) or Aβ(1–15) hydrolytic fragments is very common for MS-based PTM studies, because these peptides are more resistant to aggregation and have much more stable dissociation patterns, as compared to the full-length Aβ proteoforms.

Most of the Aβ PTMs, as well as the truncated proteoforms, can be easily distinguished by MS since they change the mass of the Aβ ions and their fragments. The study of isoforms is a special issue which may involve the use of the most advanced MS methods, such as chiral derivatized ultraperformance liquid chromatography tandem mass spectrometry (CCD-UPLC-MS/MS) [22] and ion mobility separation (IMS) for the additional separation of Aβ racemates [20]. Tandem MS with the acquisition of characteristic fragmentation spectra has been shown to be an efficient approach for estimating the isoform ratio. In particular, electron capture dissociation (ECD) fragmentation gives characteristic Aβ fragments (c6+57 and z36–57) in the presence of isoD7 [23,24,25]. Collision-induced dissociation (CID) may also be used to estimate the iso/norm content by measuring the intensity ratio of the characteristic peaks in MS/MS spectra, and can be applied even more broadly with different types of MS instruments [23,26,27,28].

Of all Aβ proteoforms, Aβ with the isomerization of D7 (isoD7-Aβ) deserves special attention since its accumulation correlates with the key pathological changes in amyloidosis: isoD7-Aβ is more prone to zinc-dependent oligomerization [29]; it exerts higher neurotoxicity and receptor toxicity in vitro [30,31]; and it is able to induce plaque formation in vivo [32]. Moreover, the selective removal of isoD7-Aβ in an AD transgenic mouse model, using monoclonal antibodies, resulted in restored cognition and a reduced plaque burden [33]. Altogether, these results point to the importance of isoD7-Aβ as a target for diagnostics and therapy. The dynamic study of isoD7 accumulation in the brain may further clarify whether its abundance in amyloid plaques correlates with the increased aggregation, or whether this is the result of spontaneous Asp-isoAsp conversions in aggregated Aβ [19].

Therefore, the dynamic accumulation of Aβ proteoforms in the brain of transgenic 5xFAD mice, simulating Aβ-plaque formation and AD progression, was studied using MALDI TOF/TOF, TIMS-TOF MS, and MS/MS. The obtained results suggest that isoD7-Aβ and other proteoforms correlate with the formation of Aβ deposits.

## 2. Results and Discussion

### 2.1. The Accumulation of Total Amyloids in the Murine Brain

**Figure 1:**
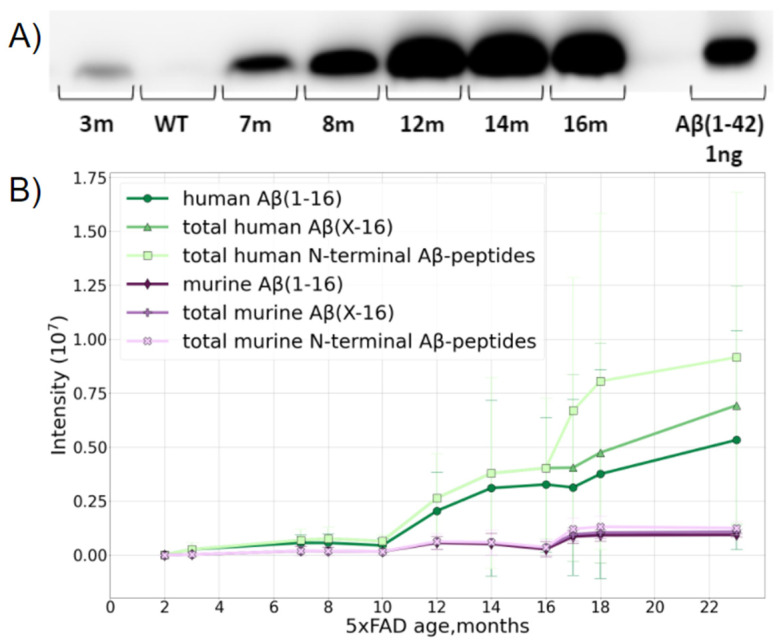
Dynamics of accumulation of Aβ in the brain of 5xFAD mice with age. (**A**) WB of Aβ extracted from 1 mg of brain tissue from mice of different ages. (**B**) Accumulation of human and murine Aβ with mouse age measured by LC-MS/MS. The accumulation and transformation of both native mouse Aβ (murine Aβ shown in purple) and transgenic human Aβ (shown in green) were monitored. Aβ (1–16) shows the changes in the intensity of a peptide starting at position 1 and ending at position 16, i.e., containing the first 16 N-terminal amino acids of Aβ. Aβ (X-16)—shows the sum of intensities of the variety of peptides starting at different positions on the N-terminus, i.e., 1, 2, 3, and so on, and all ending at position 16. The deviation of this value from the Aβ (1–16) curve demonstrates the input of the various truncated forms. Total N-terminal Aβ peptide curves show the sum of the intensities of all N-terminal peptides starting at various positions on the N-terminus, and ending at different positions on the C-end of the corresponding peptides. The deviation of these curves from the previous ones shows the accumulation and input of peptides carrying missed cleavages.

The study was carried out using transgenic 5xFAD mice, a typical model widely used for AD studies. Besides the normal production of murine Aβ, this line overexpresses the human transgenes of amyloid precursor proteins and presenilin-1 with five severe familial AD-associated mutations. This leads to an overproduction of human Aβ. The formation of fibrillar structures in the brains of these mice can be detected from 2 months of age [34]. A preliminary assessment by Western blotting (WB) suggested that the age-related accumulation of Aβ (1–42) in the brain of 5xFAD mice reaches a plateau at the age of 12 months (Figure 1A), and the maximum Aβ content is of the order of 10 ng per 1 mg of brain tissue.

The MS analysis distinguished the over-expressed human and native murine Aβ species and showed that the accumulation of both forms with age was a nonlinear process (Figure 1B). The presence of the murine form did not interfere with further analyses in any way, and, after the age of 10 months, the relative MS intensity of human Aβ became orders of magnitude higher than that of the native murine peptide form.

According to the MS data, Aβ became reliably measurable in 5xFAD mice at 2–4 months and its content slowly increased, reaching an intermediate plateau level at about 6–8 months. Between 9 and 12 months, rapid accumulation began, and while murine Aβ practically reached its maximal content at 12 months and stopped growing further, the human peptide continued to accumulate even at 16 months, though the rate of this process begins to slow down in this period (Figure 1B).

After 10 months, the input of various truncated forms became notable, and after 16 months, a jump in the number of peptides with missed cleavages was observed, probably indicating the extensive aggregation and obstruction for enzyme access. This presumption was also partially confirmed by the changes in the amounts of the peptides Aβ 17–28 and Aβ 29–42, which significantly dropped at 16 months despite Aβ accumulation (Supporting Information, Figure S1), and it was also confirmed by the small drop in the Aβ42 and total C intensities, since the long C-terminal peptides with missed cleavages have lower ionization efficiencies.

The accumulation of full-length Aβ and its Aβ (1–16) fragment demonstrated the same tendencies; thus, the use of this fragment for further analyses of isoD7 formation in Aβ is acceptable.

### 2.2. Aβ Diversity

A large variety of truncated and modified forms of Aβ were identified by the LC-MS/MS analysis using TIMS-TOF. The sequence coverage of the human and murine peptides extracted from 5xFAD mice brain samples were analyzed in dynamics and compared with the data for one human AD brain sample without dynamics (Figures S2–S4, Tables S2 and S3). The sample of the human brain extract was significantly less concentrated and showed a total Aβ level equivalent to an approximately 4- to 8-month-old mouse. The data comparison for specific forms (diversity at the C-terminus, N-terminus, and PTMs) is discussed below in more detail.

#### 2.2.1. Diversity at the C-Terminus

**Figure 2:**
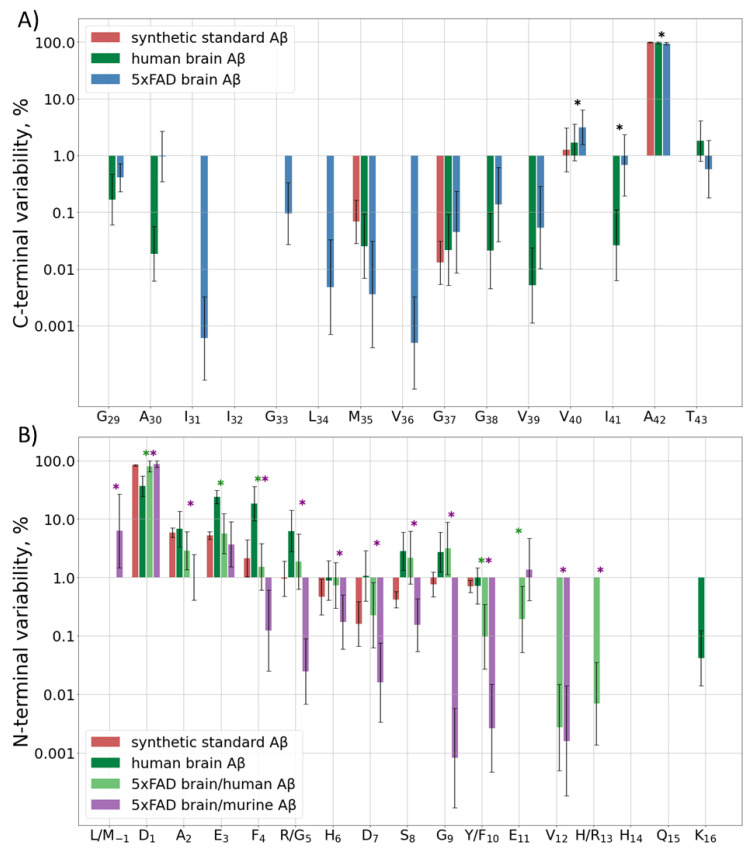
(**A**) C-terminal and (**B**) N-terminal variability of detected Aβ forms. Input of various Aβ forms ending (**A**) or starting (**B**) at each position of the sequence indicated at the horizontal axis to the total intensity of the corresponding terminal peptides for the synthetic standard, human, and murine peptide extracts from 5xFAD mice or the human AD sample. Mean values ± SD are shown, significant differences (*p* < 0.05) between human Aβ from the human and 5xFAD brain samples and between human and murine Aβ, both from the 5xFAD samples, are marked with green and purple asterisks (*), respectively. *p*-values for statistically significant/insignificant differences between groups are shown in Supplementary Table S2H,N.

The C-terminal sequence of human and murine Aβ is the same, so it is not possible to determine the origin of the C-terminal peptides observed in the hydrolyzed samples of 5xFAD brain extracts. Besides the main peptide form, i.e., Aβ42, which was observed in all samples, about 4% of Aβ40, and 1% of Aβ43 and Aβ41, were registered. Other variants of the C-terminus were detected at even lower amounts, only in the eldest mice (Figure 2A, Table S2C). The human sample showed an approximately similar distribution, but the input of Aβ40 was even lower, only at the level of 2% (Supplementary Table S2H).

#### 2.2.2. Diversity at the N-Terminal

Murine and human Aβ differ by three amino acid residues in the Aβ (1–16) sequence, so they were analyzed separately. The predominantly observed form for both murine and human Aβ peptides in most samples of the murine brain extracts started from D1 (Figure 2B, Table S2N). The only exception was observed for the murine Aβ in 2-month-old mice, for which the M(-1) form was the most abundant, probably indicating the detection of APP instead of Aβ itself. Other detected forms for both peptides started with positions 2–11, with increased cleavage around residues 3, 5, and 8–9 for the human peptide and 2–3 for the murine one (Table S2N). In order to rule out the artificial origin of these observations, the degradation and unspecific cleavage of a synthetic Aβ (1–16) peptide were also monitored. A narrow Gaussian-like dependence on the distance from the N-terminus was observed.

The truncated forms demonstrated the largest variability at 7–14 months of age. At earlier ages they were probably at or below the limit of detection and cannot be quantitated correctly, and at later stages the human Aβ was very rapidly accumulated (Table S2N).

In the human brain extracts, though Aβ (1–16) was the predominant N-terminal form, its input was only 20–60% depending on the brain region. Another 10–40% were represented by truncated forms starting at positions E3–R5, and about 4–10% started at S8 and G9. Despite the fact that the trends for increased truncation at certain positions of the human Aβ in the human and murine brain tissues are similar, the levels of magnitude are significantly different (Table S2H). The underlying mechanisms of this difference, as well as the reason for the different truncation patterns for the human and murine Aβ peptides in 5xFAD mice, require further investigation.

#### 2.2.3. Post-Translational Modifications

**Figure 3:**
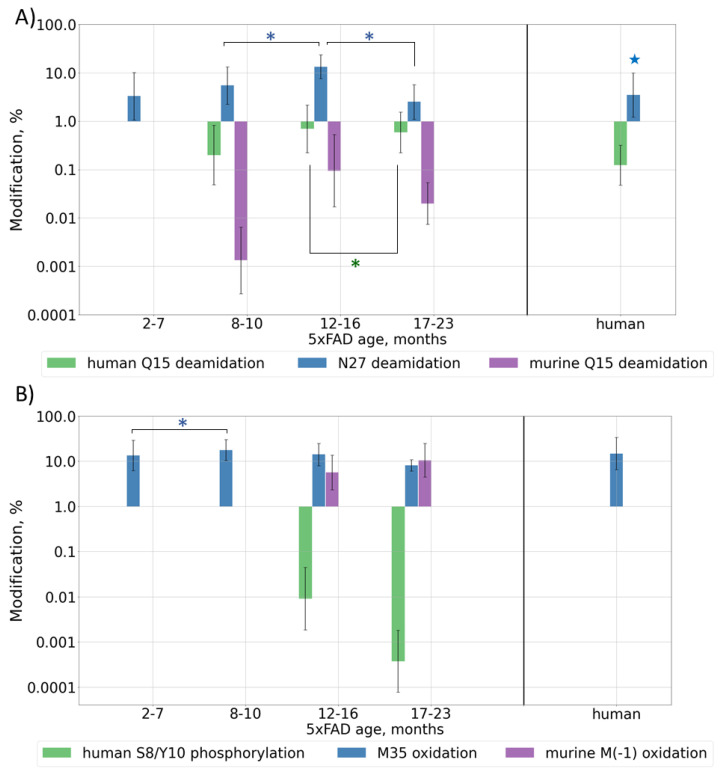

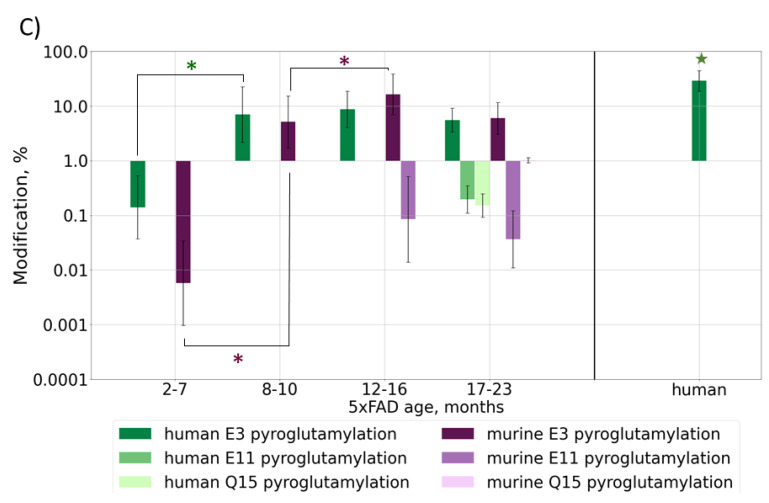
Content of various post-translationally modified (PTMs) Aβ forms in peptide extracts from 5xFAD mice of different ages or human AD sample: (**A**) deamidation of Q15 and N27; (**B**) phosphorylation of S8/Y10 and oxidation of M35, M(-1); (**C**) pyroglutamylation of E3, E11, Q15. Average values ± SD are shown. For better visualization, mice were grouped by age according to the phases of Aβ accumulation (Figure 1B). Monthly values can be found in Figure S5. *p*-values < 0.05 were considered as significant. Significant differences between age groups of mice are marked with asterisks (*). For comparison with the human AD reference brain sample, average values for mice over 6 months of age were taken (shown with stars 🟊). *p*-values can be found in Supplementary Table S3.

Deamidation is often considered as a “molecular clock” and is associated with protein aging; thus, its levels should increase with Aβ and mouse ages. Deamidation was detected for residues Q15 and N27, but, contrary to the expectations, the observed modification levels reached their maximum at approximately 12 months of age and then began to decrease, probably due to the very high accumulation rate of human Aβ at later ages (Figure 3A and Figure S5A, Table S3). Presumably, if the lifespan of the mice were long enough, this value would probably start to increase again when the human form would reach its stable maximum content, similar to what the murine form does, but currently this cannot be checked. Deamidation for the human brain sample showed a level similar to that of an average adult mouse (0.1% (*p*-value = 0.2) and 3% (*p*-value = 0.02) for sites Q15 and N27, respectively) (Table S3).

Since oxidative stress was shown to play a pivotal role in the pathological brain alterations in AD [35], the levels of oxidative modifications are also expected to grow with Aβ accumulation in mouse brain tissues, i.e., with mouse aging. The oxidation of Met35 was observed across all ages and, unlike deamidation, its level did not demonstrate such a pronounced decrease after the beginning of the rapid growth of human Aβ at 12 months, and in general, it correlates with the total amyloid accumulation. The average observed level of oxidation was approximately 13%. The human brain sample showed a similar oxidation level of 15% (*p*-value = 0.2). For murine Aβ, a significant amount (up to 18%) of M(-1) oxidation was also observed in older mice (Figure 3B and Figure S5B, Table S3).

The formation of a pyroglutamate is another often reported modification of Aβ truncated at position E3, which is also considered to be important for the mechanisms of plaque formation [36]. This modification was observed at approximately the same level, 6–8%, for both murine and transgenic peptides in 5xFAD samples, and it followed the same general behavior pattern. This involves the modification becoming stably registered at approximately 7 months, then growing to the maximal level followed by a drop down as the rapid accumulation of the transgenic peptide interferes. At other sites, this modification was observed for mice over 16 months of age when the number of peptides with missed cleavages was significantly increased, probably indicating the accumulation of degraded and damaged peptides and overall oxidative stress (Figure 3C and Figure S5C, Table S3). In the human brain sample, the level of pyro-Glu E3 was approximately 3–4 times higher than that in mice (*p*-value = 7 × 10^−7^), but this was probably not due to a difference in the modification rates or mechanisms, but rather in the truncation process, since this value correlates well with the 3–4 times higher abundance of the truncated peptides at this position (*p*-value = 1.1 × 10^−6^) (Figure 2B).

The phosphorylation of Aβ was shown earlier only by WB [37], but was never confirmed by MS. During the database search, the phosphorylation of S, T, and Y residues was allowed as a variable modification, and several spectra were assigned to the phosphorylated Aβ peptides (Figures S2–S4 and Figure 3B, Table S3). The modification was shown to be located at residues S8 and Y10; however, a more thorough analysis of the fragmentation spectra did not allow one to reliably confirm or disclaim the presence and position of this PTM (Figure S6). Moreover, a doubly modified peptide form was identified. Besides this, two fragmentation spectra were assigned to peptides with phosphorylation at position S26, but the overall quality of these spectra was not high enough for a confident report and remains a question to be explored. Similarly, in 5xFAD mice, we observed an almost complete phosphorylation at site T719, while this modification at T714 seemed to be a positioning error. This site was not reported earlier at UniProtKB. Positions 729, 730, and 743, which were listed as possibly modified, were not covered reliably enough to make conclusions on their modification state and no peptides phosphorylated at site Y757 were detected (Figures S2 and S3). This modification was not detected in the human brain extract at most of these sites (Figure S4) and the reliability of the several assigned peptide–spectrum matches was not confident enough, but this is probably due to the lower concentration of Aβ peptides. Therefore, the use of selective phosphorylated peptide enrichment methods is necessary for further research to confidently evaluate the presence, position, and level of this modification.

**Figure 4:**
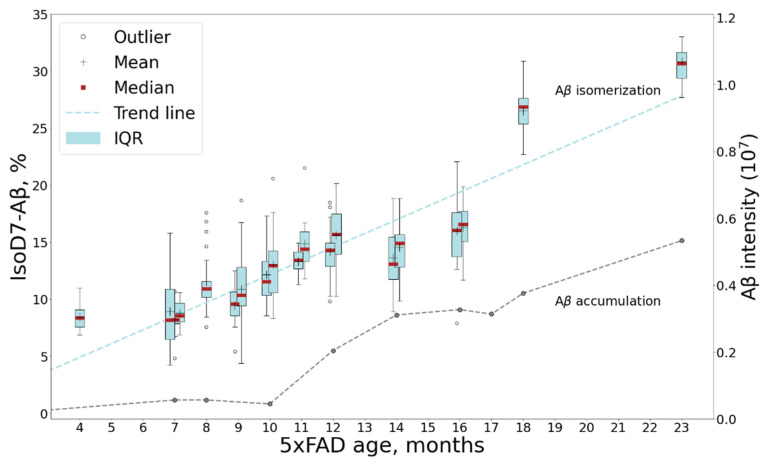
The dynamics of isoD7-Aβ content in 5xFAD mice brain samples studied by MALDI TOF/TOF. The boxplot graph shows the changes in the isoD7-Aβ content in samples from mice of different ages. For timescale comparison accumulation of human Aβ with mouse age measured by LC-MS/MS (see Figure 1) is also shown. Average values for combined mouse age groups and significance estimations can be found in Figure S7.

Isomerization of D7 is considered by some researchers as one of the key events for Aβ aggregation and accumulation onset; thus, the changes in its levels with mouse age was of special interest. The relative content of isoD7-Aβ was estimated by measuring the ratio of the intensities of the marker (b6) and “base” (b11) fragments in the MALDI-TOF MS/MS spectra of Aβ (1–16) fragments (see Section 3). The average intensity ratio of these fragments was calculated from 20 accumulated MS/MS spectra of each sample (Supplementary Table S1). The obtained dynamics of isoD7-Aβ accumulation is shown in Figure 4.

Despite the rather large value of the standard deviation for the elements of the cohort, the tendency towards an increase in the fraction of the isomerized form is beyond doubt, and this trend is confirmed by a high Kendall coefficient of 0.88, as well as a statistically significant *p*-value (1.06 × 10^−7^).

An interesting nuance, which should be especially noted, is that a more thorough comparison of the accumulation dynamics for the isomerized form and the amyloid in total reveals a lag of a few months between each (Figure 4). The increase in the relative content of isoD7-Aβ appears to begin after 7 months of age, while no increase in the content of the amyloid in total is observed at this time. This suggests that progressive isomerization may trigger the subsequent phase of a sharp increase in the total Aβ content, which begins after 9 months of age. It is noteworthy that the first behavioral disorders are detected precisely in this age period [38]. However, in general, the growth rate of the isomerized form over the lifespan of a mouse appears to be more or less constant (Figure 4 and Figure S7), what may reflect the spontaneous isomerization process accommodating the aging of amyloid deposits. The obtained results are in good agreement with the earlier published qualitative study of 5xFAD brains, which revealed a tendency towards the accumulation of isoD7-Aβ with age between 3 to 12 months, with the appearance of the first iso-containing deposits between 3 and 6 months of age [33].

**Figure 5:**
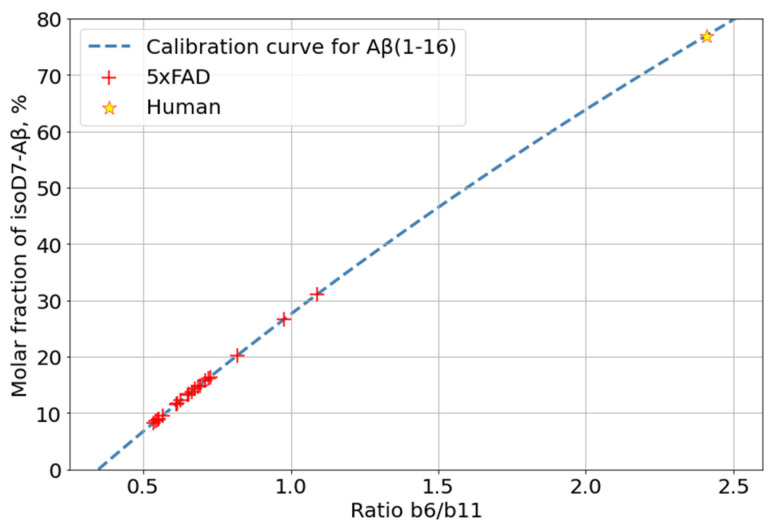
Comparison of isoD7-Aβ content measured for 5xFAD and human AD brain samples.

In the human AD patient, the isoD7-Aβ content, according to our measurements, turned out to be approximately 76%. This is consistent with the results of previous studies [17,18,20,22]. Thus, the maximal level of isoD7-Aβ in 5xFAD mice is much lower than that in human AD brain tissues (Figure 5). This is probably due to the short lifespan of mice and the insufficient time for both the total Aβ and its isomerized forms to reach the kinetically stable plateau levels.

## 3. Materials and Methods

### 3.1. Reagents and Peptides

All chemicals and solvents used throughout this study were of HPLC-grade or better and were obtained from Sigma-Aldrich (St. Louis, MO, USA). The normal and isoD7-containing synthetic Aβ peptides (Aβ (1–16), isoD7-Aβ (1–16), Aβ (1–42), and isoD7-Aβ (1–42)) were obtained by solid-phase synthesis and were purified to a purity of more than 99.5% using HPLC (BioPeptide Inc., San Diego, CA, USA) and then were dissolved in 10% acetonitrile to 0.5 mg/mL, aliquoted (50 μL), and stored at −80 °C.

### 3.2. Animals

A transgenic mouse line 5xFAD from the laboratory of Jackson, USA (Stock Number: 006554) [34] was used. This line has 5 severe familial AD associated mutations in genes of the human amyloid precursor protein (APP) (SweK670N, M671L, LonV717I, and FloI716V) and presenilin 1 (PSEN1) (M146L and L286V), expressed under the control of the murine Thy1 promotor. Thus, these mice express their own native murine Aβ at a normal rate and transgenic human Aβ at a much higher level. The increased accumulation of human Aβ (1–42) results in accelerated impaired cognitive functions of the brain. Transgenic 5xFAD mice start to show the first behavioral disturbances at around 6 months of age, which become most pronounced by 9 months [38]. The used cohort consisted of 24 animals: two mice each at 2, 8, 9, 10, 12, 14, and 16 months of age; three animals at 3 and 7 months of age; and one mouse each at 4, 17, 18, and 23 months of age.

The animals were kept in a colony with access to food and water ad libitum in a room with a controlled temperature of 20 ± 2 °C and a humidity of 40–60% at a 12-h light/dark cycle. Experiments with the mice were performed in accordance with the procedures approved by the local ethics committee and animal control authorities (Guidelines for accommodation and care of animals. Environment, housing and management, 2016).

All 5xFAD mice were genotyped to confirm the presence of all mutations. For this, DNA was isolated from a part of each animal’s tail, which was placed in 300 μL of the lysis buffer (10 mM Tris-HCl pH 8, 100 mM NaCl), containing proteinase K, incubated at +55 °C for 16–20 h with a rotation at 650 rpm, and then stirred and centrifuged for 5 min at 14,000× *g* rpm at room temperature. The top fraction containing DNA was further washed once with isopropanol and twice with 80% ethanol, followed by centrifugation for 15 min at 14,000 rpm at +4 °C. The presence of the transgenic (human) PSEN1 and APP genes was tested by a polymerase chain reaction (PCR) using the insert-specific primers 5′AAT AGA GAA CGG CAG GAG CA3′ and 5′GCC AT G AGG GCACT AAT CAT3 (for PSEN1), and 5′AGG ACT GAC CACT CG ACC AG3′ and 5′CGG GGG T CT AGT T CT GCA T3′ for APP [39]. The control primers were 5′CT A GGC CAC AGA AT T GAA AGA T CT3′ and 5′GT A GGT GGA AAT T CT AGC AT C C3′.

### 3.3. The Extraction and Hydrolysis of Aβ Peptides

Aβ peptides were isolated from brain tissues using FA extraction, immunoprecipitation, and SPE (solid phase extraction) approaches. Each mouse was prepared at least twice, and each sample was run at least in duplicate. Murine brain samples were homogenized on ice using Potter’s glass tissue grinder in four volumes of a lysis buffer (20 mM Tris, 2.5 mM EDTA, 137 mM NaCl, pH 7.6) containing a protease inhibitor cocktail (Roche). After adding FA to a final concentration of 70%, the samples were sonicated (10 min × 2), vortexed, and centrifuged (30,000× *g*, 1 h, +4 °C). FA from the supernatant was evaporated using a centrifugal vacuum concentrator (Eppendorf). The obtained acidic extracts were stored at −80 °C.

The SPE of Aβ was performed using Oasis MCX cartridges largely according to the published protocol [40] with some modifications. Acidic extracts were dissolved in 8M/2M urea/thiourea, and H_3_PO_4_ was added up to 2%. The SPE cartridges were preconditioned with 1 mL MeOH and equilibrated with 1 mL 4% H_3_PO_4_. Two mL of the sample was loaded onto the SPE cartridge and washed with 2 mL of 4% H_3_PO_4_ and 10% ACN. The Aβ-enriched fraction was eluted with 1 mL of 75:15:10 (*v*/*v*/*v*) ACN/H_2_O/NH_4_OH solution and samples were evaporated in a centrifugal vacuum concentrator to 100 μL.

For the quantitative MS analysis, samples were hydrolyzed by LysC protease (Promega) to obtain hydrophilic Aβ(X-16) fragments (Figure S8). Samples after the SPE (20 μL) were mixed 1:1 with 100 mM ammonium bicarbonate, 0.4 μg of LysC, and then incubated at 37 °C for 4 h.

### 3.4. Western Blot

The obtained SPE eluates were treated with 2× sample buffer, separated by 12% SDS-PAGE using the Tris-Tricine buffer system [41], and electroblotted to a nitrocellulose membrane. The membrane was blocked with a 2.5% milk solution and incubated with primary antibodies 6E10 (Biolegend, San Diego, CA, USA, 1:8000) overnight. HRP-conjugated goat antimouse IgG (Hy-test, 1:5000) were used as the second antibody with an incubation of 1 h. The resulting bands were developed using the SuperSignal chemiluminiscent substrate (Thermo Fisher Scientific, Waltham, MA, USA). Images were collected using the SYNGENE G:Box System (Syngene, Cambridge, UK).

### 3.5. Human Samples

The human brain tissue of an 82-year-old sporadic AD patient was collected after an informed consent of the next of kin, at the time of autopsy, with a post-mortem delay of about 15 h. Samples of the temporal lobe and hippocampus were either flash-frozen in liquid nitrogen for further MS analyses or fixed in 10% buffered formalin (BioVitrum, Saint-Petersburg, Russia) and embedded in paraffin for histological analyses. In total, eight separate tissue samples were subjected to an MS analysis.

Histological sections of 4–5 μm thick paraffin-embedded brain tissue was produced and stained with Congo red (BioVitrum, Russia). Antibodies to tau proteins and Aβ were used for immunohistochemical (IHC) staining.

The histological slices were dewaxed in three changes of xylene and alcohols of decreasing concentrations. Antigen retrieval was performed by boiling of slices for 20 min in a citrate buffer with 0.05% Tween 20 and a pH = 6.0 in a microwave oven. The slices were washed twice in PBS (Biolot, Saint-Petersburg, Russia). To inactivate the endogenous peroxidase, a 3% hydrogen peroxide solution was applied for 20 min and then washed off twice in PBS. Nonspecific binding was blocked by incubation in 1% bovine serum albumin/PBS for 20 min. Primary antibodies (mouse-anti-τ T5530, Sigma-Aldrich; rabbit-anti-beta amyloid ab201060, Abcam, Cambridge, UK) were diluted in a blocking buffer of 1/500, applied to the sections placed in a wet chamber, and incubated overnight at +4 °C. The slices were incubated with peroxidase-labeled secondary antibodies (donkey-anti-rabbit IgG (H+L) highly cross-adsorbed secondary antibody, HRP, A16035, Invitrogen dilution 1/500; goat anti-mouse IgG H&L (HRP), ab6789, Abcam dilution 1/500) for an hour at room temperature in a humid chamber. Antibody binding was detected by the reaction of horseradish peroxidase with 3,3′-diaminobenzidine (DAB Substrate Kit, ab64238, Abcam, Cambridge, UK). Cell nuclei were stained with Mayer’s hematoxylin. Histological analyses confirmed the presence of AD neuropathology, including abundant senile plaques and tau tangles in the temporal lobe and hippocampus (Figure S9).

### 3.6. MALDI-TOF/TOF MS

A quantitative isoD7 analysis was performed using a MALDI TOF/TOF UltrafleXtreme mass spectrometer (Bruker Daltonics, Bremen, Germany) equipped with a 355-nm Nd:YAG laser operating in pulse mode with a frequency of up to 1 kHz and forming a beam using the smartbeam2 technology. AnchorChip™ 800 384-point targets were used for sample application. Fragments of β-amyloid 1–16 were analyzed using α-cyano-4-hydroxycinnamic acid (HCCA) as a matrix. Samples were applied using the dried droplet method, where drops of the sample solution and matrix substance were mixed on the target, then dried with air. Fragmentation was carried out using the laser ionization fragmentation technology (LIFT) in CID mode with argon as the collision gas.

The previously described label-free method, based on the analysis of intensities of characteristic peaks in the MS/MS spectra of Aβ, was used to quantify the percentage of isomerized peptides in analyzed samples [26]. The fragmentation of the modified peptide in CID mode does not result in the production of fragments strictly for isomerization; however, the higher efficiency of peptide bond breakage near the isoAsp (as compared to the native Asp) residue results in an increase in the intensity of the corresponding fragments (b6, b7, and y10 for Aβ (1–16)) [42]. Based on this observation, calibration curves for the changes in the relative intensities of such peaks (the ratio of the intensities of the marker peaks compared to “base” ones, i.e., those not affected by isomerization (for example, b11 andy11 for Aβ (1–16)) depending on the isoAsp content were measured and used to estimate the proportion of the isoD7-carrying Aβ form, using MS/MS spectra, of the peptide ions (Figures S10 and S11).

Calibration curves for isoD7 Aβ were built using synthetic standards of both native and isoD7-containing forms of Aβ (1–16). The two peptide forms were mixed to obtain a set of binary mixtures in which the percentage of the isoD7 Aβ varied from 0% to 100% in 10% increments.

To test the stability of the calibration curves in case of “complex” samples, a set of peptide mixtures with different contents of isoD7 (as described above) was spiked into fractions derived from a WT mouse brain before and after SPE, followed by LysC hydrolysis. Matrix effects did not significantly affect the shape of the calibration curve, which is what implies the applicability of this method to biological samples (Figure S12).

MALDI-TOF data was initially processed using the FlexAnalysis 3.3 software (Bruker Daltonics, Bremen, Germany). A subsequent analysis was performed using home-written Python scripts.

### 3.7. The LC-MS/MS Analysis by TIMS-TOF

All samples were analyzed in duplicate on a nano-HPLC (high-performance liquid chromatography) Dionex Ultimate 3000 system (Thermo Fisher Scientific, Waltham, MA, USA) coupled to a TIMS-TOF Pro (Bruker Daltonics, Bremen, Germany) mass spectrometer. The sample volume loaded was 1 μL per injection. HPLC separation was carried out using a packed emitter column (C18, 25 cm × 75 μm 1.6 μm) (Ion Optics, Fitzroy, VIC, Australia), with gradient elution. The mobile phase A was 0.1% FA in water; mobile phase B was 0.1% FA in acetonitrile. LC separations were performed at a flow of 400 nL/min using a 60 min linear gradient from 2% to 22% in solvent B, followed by a 10-min LC column wash step with 95% of solvent B, and a 10 min equilibration with 2% of solvent B.

MS measurements were carried out using the PASEF acquisition method. The electrospray ionization (ESI) source settings were as follows: 1500 V capillary voltage, 500 V end plate offset, and 3.0 L/min of dry gas at a temperature of 180 °C. The measurements were carried out over the m/z range from 100 to 1700. The range of ion mobilities included values from 0.6 to 1.6 V s/cm^2^ (1/k0). The total cycle time was set to 1.16 s, and the number of PASEF MS/MS scans was set to 10.

The analysis of the raw TIMS-TOF data (peak integration and visual verification) was performed using data analysis software ver. 5.3 (Bruker Daltonics, Bremen, Germany) and home written Matlab Scripts.

The identification and quantitative analysis of the variability of detected Aβ proteoforms (truncations and PTMs) were carried out using the PEAKS XPro software (BSI, North Waterloo, ON, Canada). A semispecific enzyme restriction search was conducted across a UniprotKB human or mouse database with an addition of 5xFAD transgene sequences with deamidation (NQ), oxidation (M), phosphorylation (S,T,Y), and pyro-glutamate formation (E,Q) as possible modifications. A maximum of three variable modifications per peptide was allowed, and an Ascore threshold of 20 was set for the PTMs. Mass error tolerance was set to 30 ppm for parent ions and 0.05 Da for the fragment ions. FDR thresholds were set to 0.1% at the PSM level and 1% at the protein level, with the requirement of at least one unique and one significant peptide identification.

### 3.8. Statistical Analysis

The statistical analysis of the results was performed using Python 3.7 tools. The following parameters of the distribution of measurements were estimated: means, medians, interquartile range, standard deviations, and *p*-value. To confirm the dependence of isoD7-Aβ accumulation with increasing age (see Section 2.2.3), the Kendall rank correlation coefficient was calculated. The outliers were detected based on a z-score (>3). Statistical significance for the data presented was assessed using the Mann–Whitney U-test (with a threshold *p*-value < 0.05) (Tables S2C,N,H and S3).

## 4. Conclusions

The obtained results show the non-linear dynamics of the accumulation of Aβ in 5xFAD mice with age. The concentration of Aβ in a reference human AD brain sample was at the level of a 4–8-month-old mouse. The diversity of the observed forms for both murine and human Aβ variants was mostly similar, with a significant difference in the truncation levels at position E3, and the pyroglutamate formation, respectively. Deamidation and oxidative stress levels were at similar levels. The isomerization of D7 was one of the dominant modifications of the content, which grows with approximately the same tendency from the early stages of Aβ accumulation to old age and is likely to be an important effector of the enhanced amyloid deposition that begins after 10 months of age. The obtained results suggest that particular Aβ modifications may be essential for the enhanced formation of amyloid deposits in the brains of 5xFAD mice, simulating AD progression. Although the mouse model cannot be a complete analogue of the processes occurring in the human brain in AD, this dynamic study clarifies one of the possible mechanisms that can trigger amyloidosis in AD. Importantly, the obtained data confirms the hypothesis about the relationship between the accumulation of isoD7-Aβ and the progression of AD-like pathology.

## Supplementary Materials

The following are available online at https://www.mdpi.com/article/10.3390/ijms23010027/s1.
