# Supplementary material for: The Dynamics of β-Amyloid Proteoforms Accumulation in the Brain of a 5xFAD Mouse Model of Alzheimer’s Disease
Source: Int J Mol Sci. 2021 Dec 21;23(1):27. doi: 10.3390/ijms23010027 (PMC8745018; doi:10.3390/ijms23010027)
Supplement: Supplementary file 1 [file ijms-23-00027-s001.zip › ! Suppl_files_ijms-1493189_rev2_16Dec.pdf]

Supporting Information

Article

The Dynamics of β-Amyloid Proteoforms Accumulation in the Brain of a 5xFAD Mouse Model of Alzheimer's Disease

Anna E. Bugrova [1], Polina A. Strelnikova [1,2], Maria I. Indeykina [1,3], Alexey S. Kononikhin [3,4], Natalia V. Zakharova [1], Alexander G. Brzhozovskiy [4], Evgeny P. Barykin [3], Stanislav I. Pekov [2,3,4], Maria S. Gavrish [5], Alexey A. Babaev [5], Anna M. Kosyreva [6], Anna Y. Morozova [7,8], Daniil A. Degterev [3,9], Vladimir A. Mitkevich [3], Igor A. Popov [2,10], Alexander A. Makarov [3,*] and Evgeny N. Nikolaev [4,*]

1 Emanuel Institute of Biochemical Physics, Russian Academy of Science, 119334 Moscow, Russia; anna.bugrova@gmail.com (A.E.B.); pauline.strel@gmail.com (P.A.S.); mariind@yandex.ru (M.I.I.);
nvzakharova@yandex.ru (N.V.Z.)
2 Moscow Institute of Physics and Technology, 141701 Dolgoprudny, Russia; stanislav.pekov@forwe.ru (S.I.P.); hexapole@gmail.com (I.A.P.)
3 Engelhardt Institute of Molecular Biology, Russian Academy of Science, 119991 Moscow, Russia; a.kononikhin@skoltech.ru (A.S.K.); eugbar96@gmail.com (E.P.B.); daniil_degterev@mail.ru (D.A.D.);
mitkevich@gmail.com (V.A.M.)
4 Skolkovo Institute of Science and Technology, 121205 Moscow; Russia; agb.imbp@gmail.com
5 Institute of Neuroscience, Lobachevsky State University of Nizhny Novgorod, 603022 Nizhny Novgorod, Russia; mary_gavrish@mail.ru (M.S.G.); alexisbabaev@list.ru (A.A.B.)
6 Research Institute of Human Morphology, 117418 Moscow, Russia; kosyreva.a@list.ru
7 Department of Basic and Applied Neurobiology, Federal Medical Research Center for Psychiatry and
Narcology, 119034 Moscow, Russia; hakurate77@gmail.com
8 Mental-Health Clinic No. 1 Named after N.A. Alexeev of Moscow Healthcare Department, 117152 Moscow, Russia
9 Neurological Department, Loginov Moscow Clinical Scientific Center, 111123 Moscow, Russia
10 V.L. Talrose Institute for Energy Problems of Chemical Physics, N.N. Semenov Federal Center of Chemical Physic, Russian Academy of Sciences, 119334 Moscow, Russia
* Correspondence: aamakarov@eimb.ru (A.A.M.); e.nikolaev@skoltech.ru (E.N.N.)

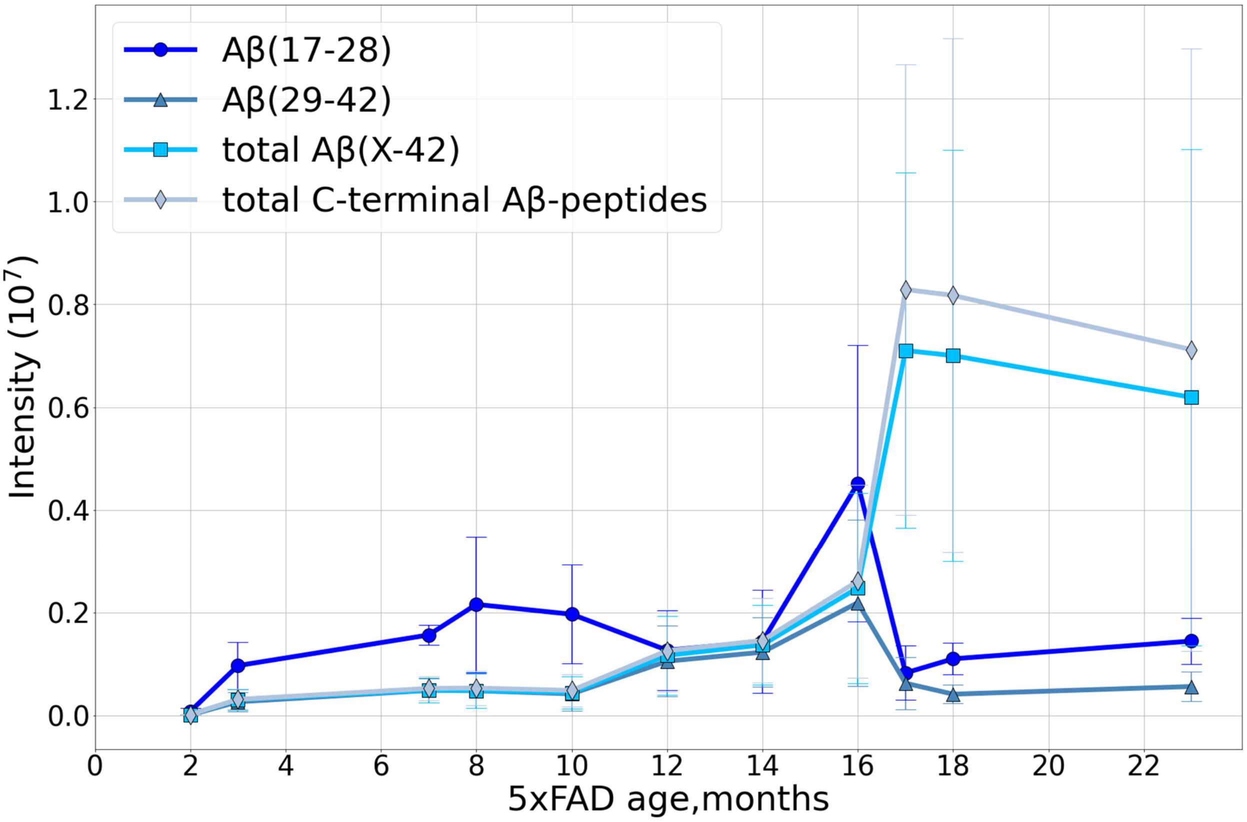

Figure S1: Dynamics of accumulation of human and murine Aβ with mouse age measured by LC-MS/MS. The accumulation and transformation of both native mouse Aβ (murine Aβ) and transgenic human Aβ were monitored. Aβ(17-28) and Aβ(29-42) show the changes in the intensities of peptide starting and ending at the indicated positions of the sequence. Aβ(X-42) – shows the sum of intensities of the variety of peptides starting at different positions and all ending at position 42. Total C-terminal Aβ peptides curve shows the sum of the intensities of all C-terminal peptides with various ends.

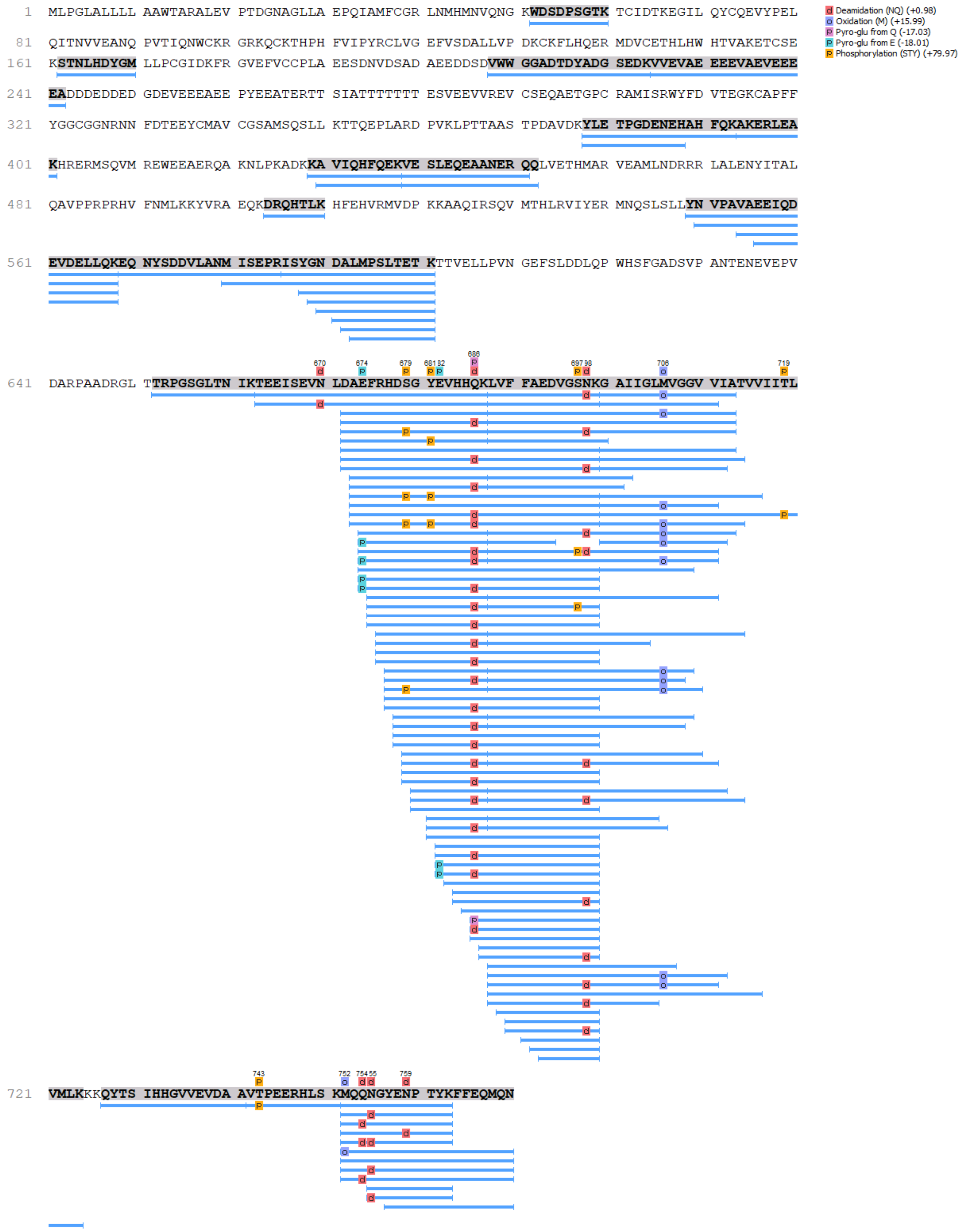

Figure S2. The sequence coverage for human Aβ extracted from brain samples of 5xFAD mice.

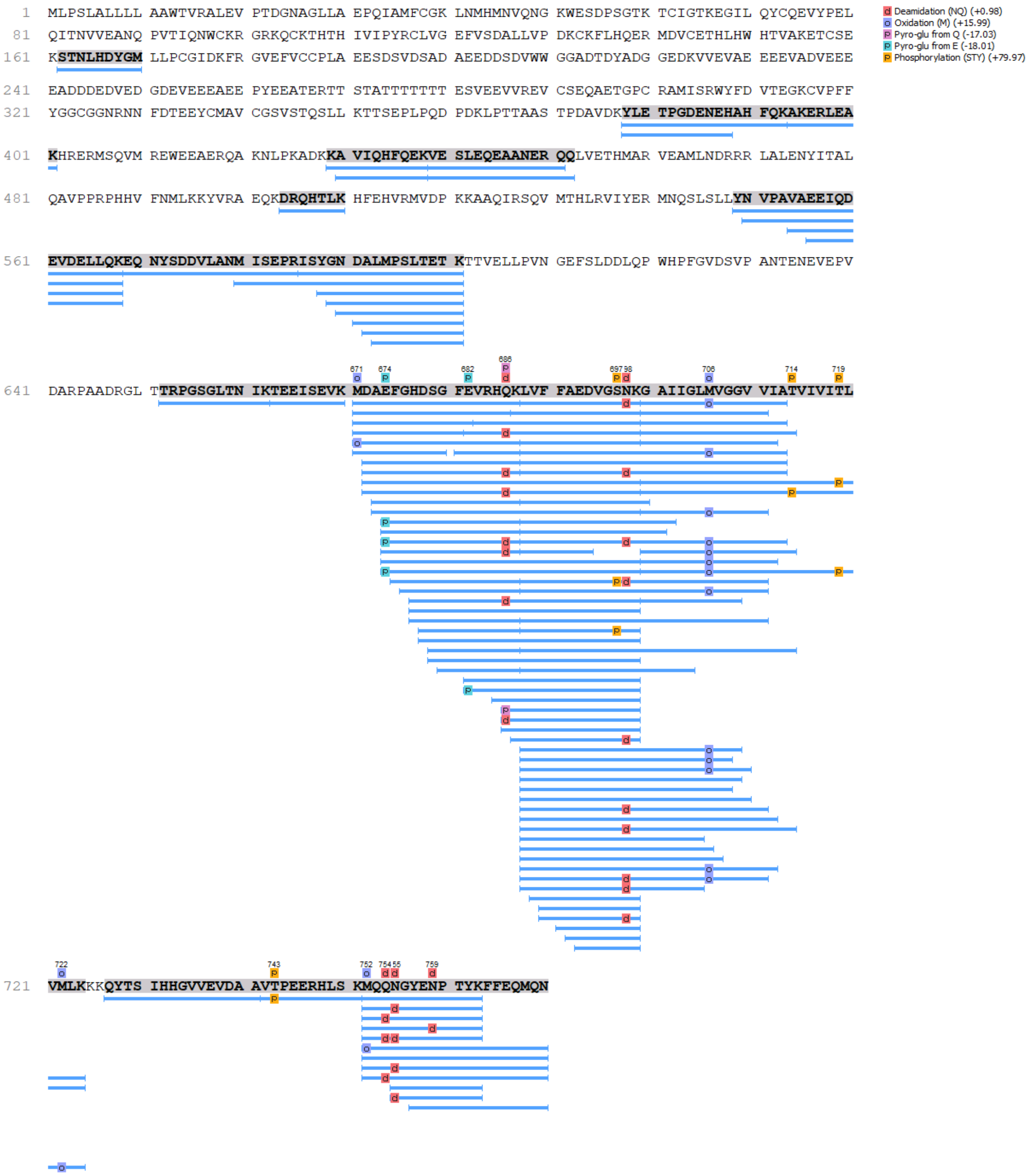

Figure S3. The sequence coverage for murine Aβ extracted from brain samples of 5xFAD mice.

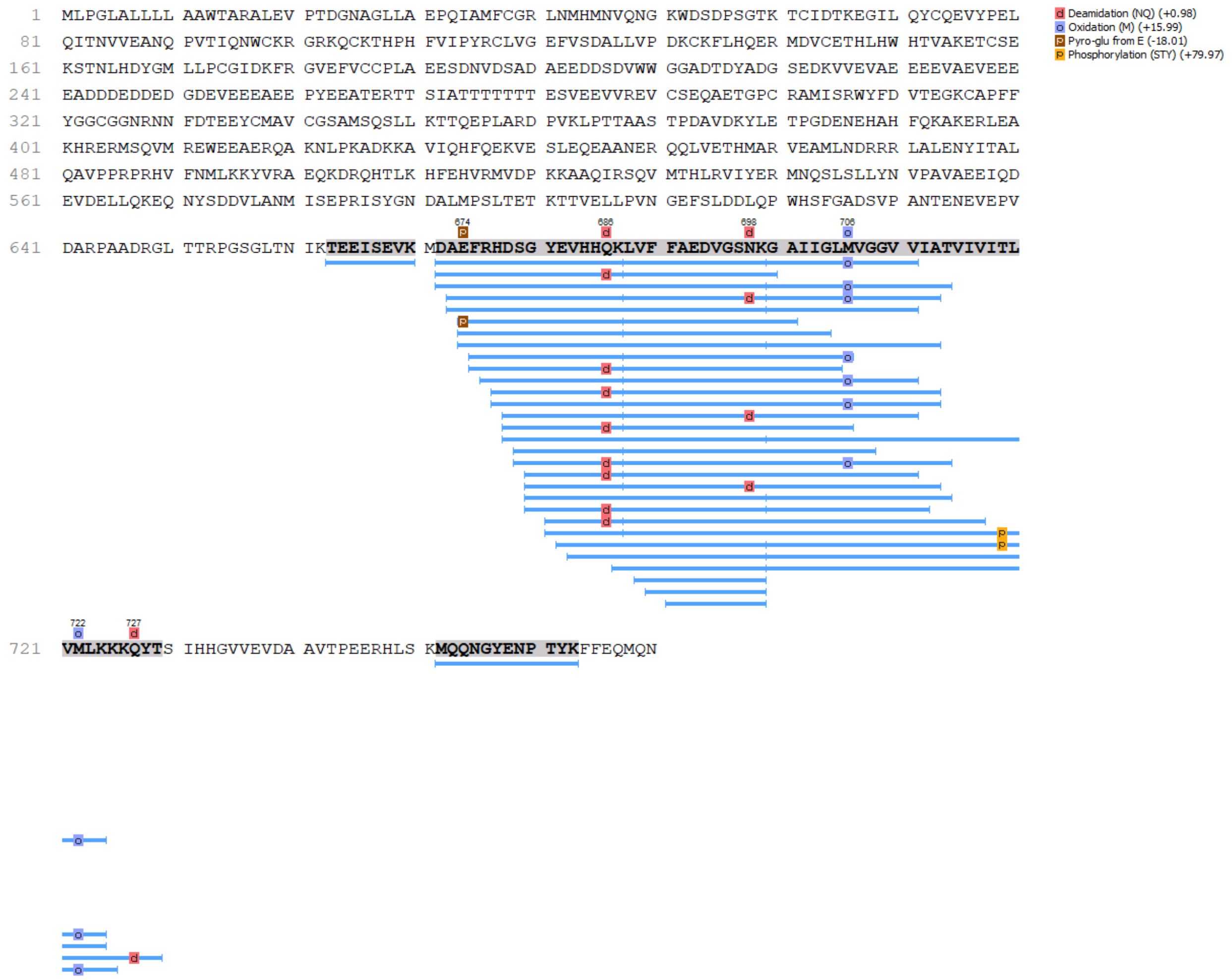

Figure S4. The sequence coverage for Aβ extracted from the human AD brain sample.

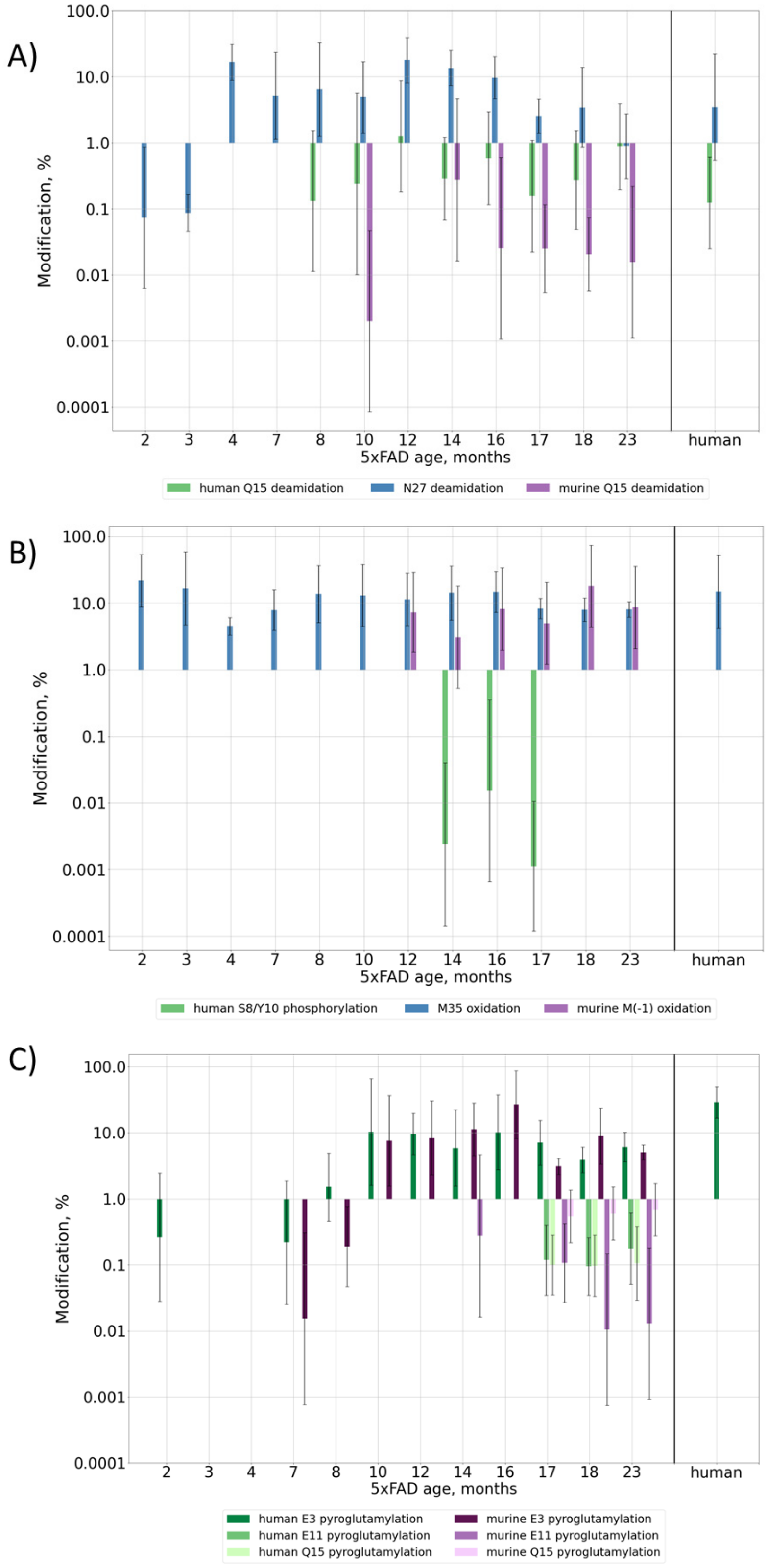

Figure S5. Content of various post-translationally modified (PTMs) Aβ forms extracted from brain samples of 5xFAD mice of different ages (by month) or human AD sample: A) - Deamidation of Q15 and N27; B) - Phosphorylation S8/Y10 and Oxidation M35, M(-1); C) – pyroglutamylation E3, E11, Q15. Average values ± SD are shown.

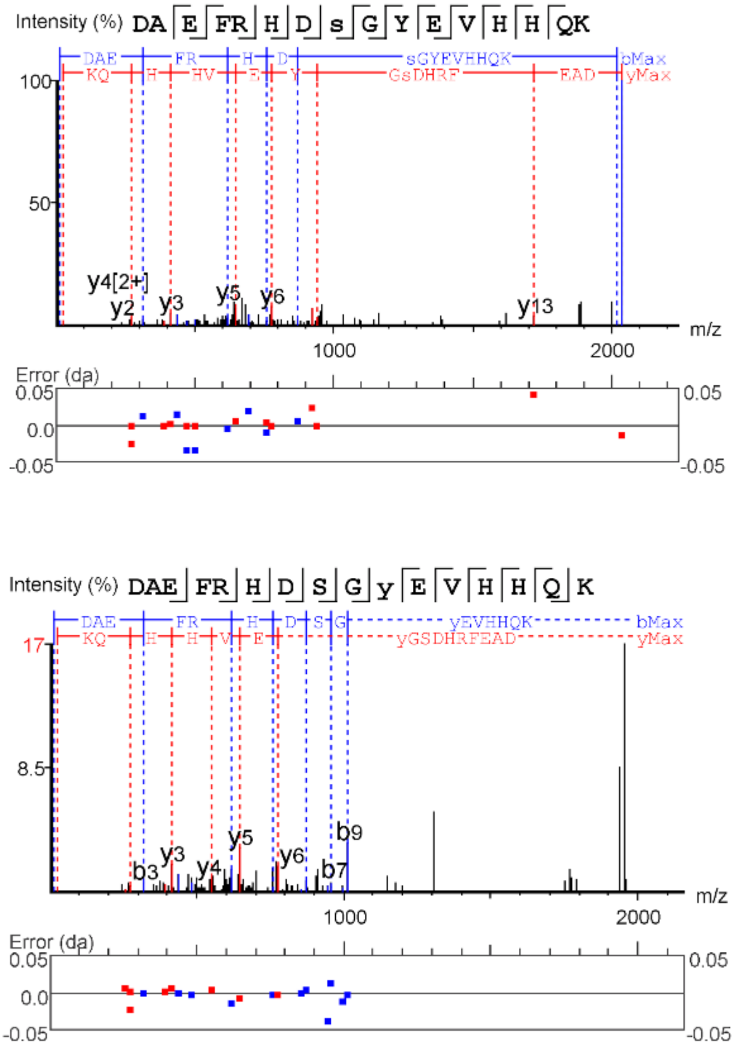

Figure S6. Observed MS/MS fragmentation spectra of phosphorylated human Aβ(1-16) with different localization sites for the modification: S8 – top spectrum; Y10 – bottom spectrum.

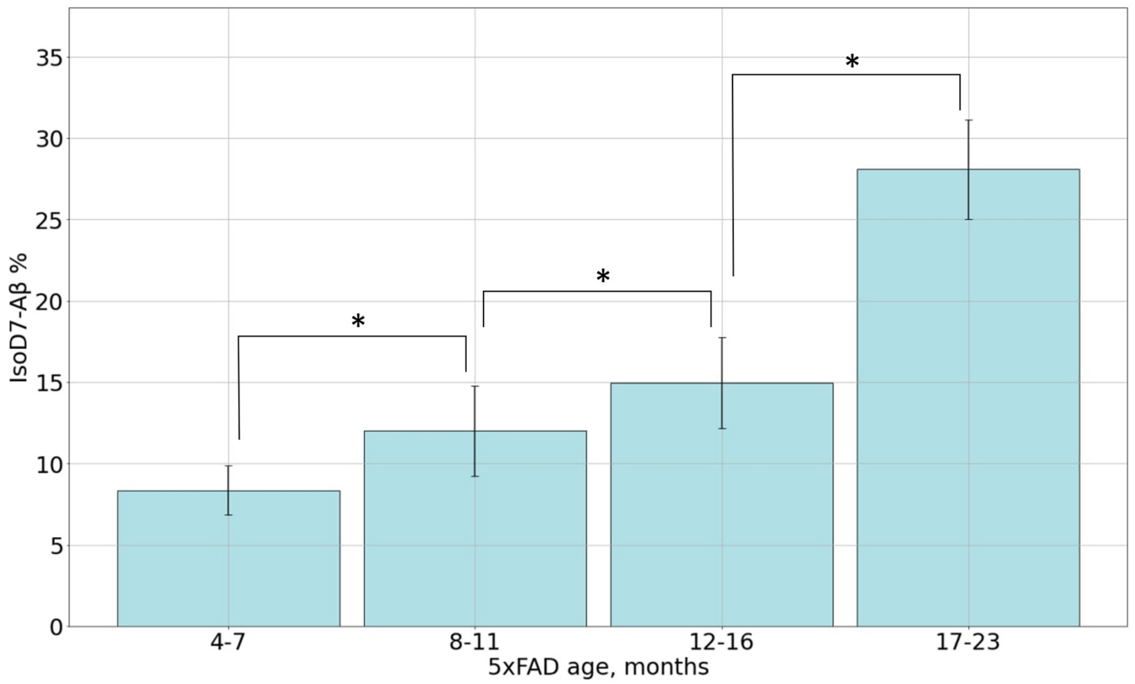

Figure S7. Iso-D7-Aβ content in brain samples of 5xFAD mice studied by MALDI TOF/TOF depending on mouse age. The mice were grouped by age according to the Aβ accumulation phases (main text fig.1, 4). Mean values ± SD are shown. Significant differences (p-value<0.05) are shown by asterisks (*).

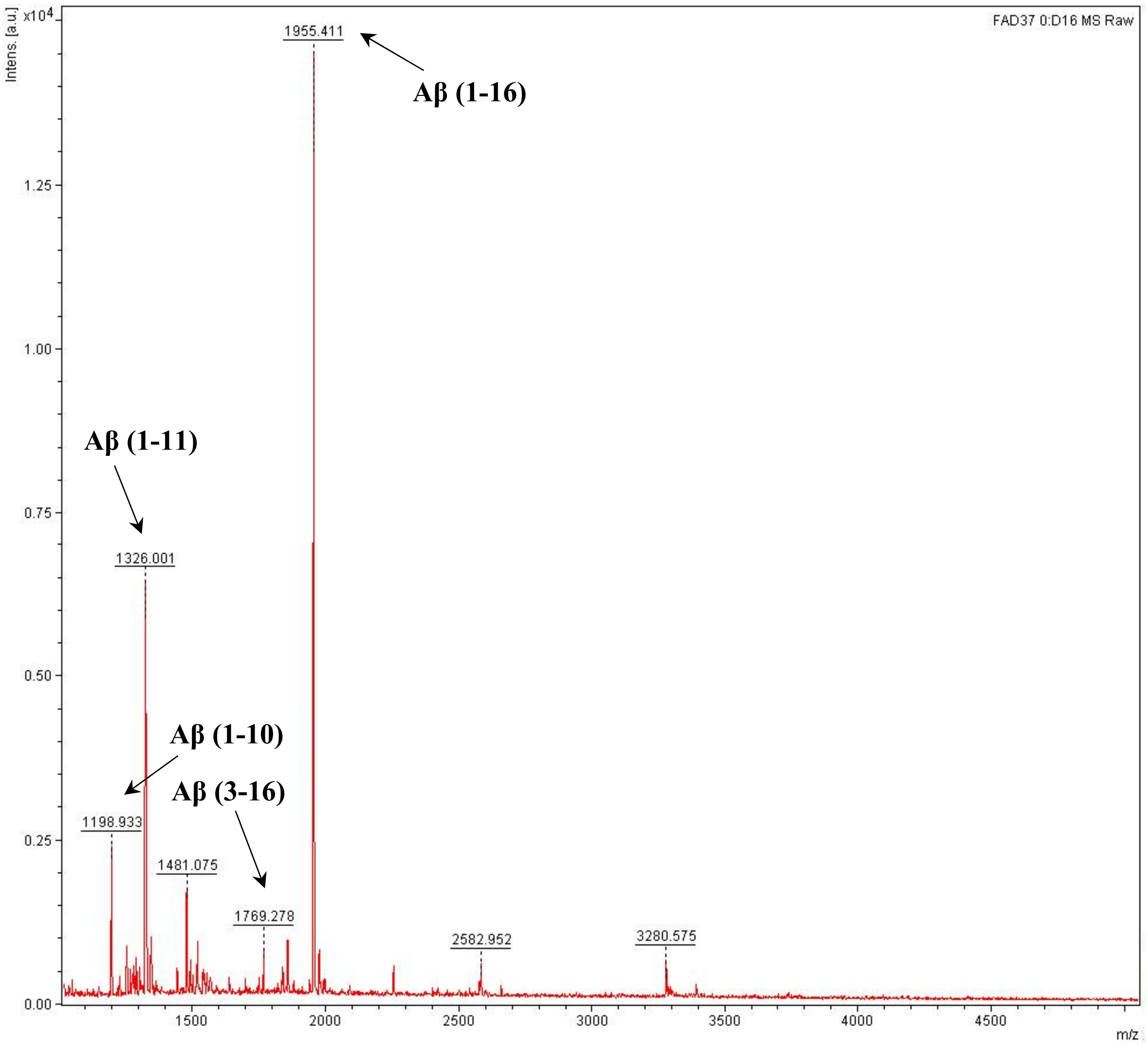

Figure S8. MALDI TOF mass spectrum of Aβ(X-16) fragments after SPE and LysC hydrolysis. Fragments annotation:

m/z 1955: DAEFRHDSGYEVHHQK Ab1-16
m/z 1326: DAEFRHDSGYE Ab1-11
m/z 1198: DAEFRHDSGY Ab1-10
m/z 1769: EFRHDSGYEVHHQK Ab3-16

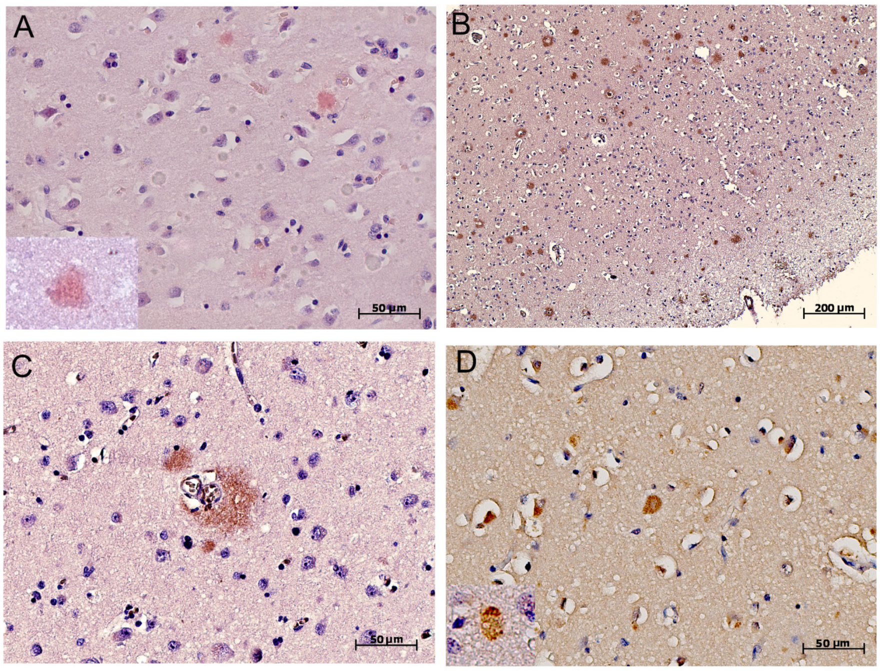

Figure S9. Neuropathological signs of Alzheimer's disease. A – Aβ plaques in the temporal cortex and a single Aβ plaque at higher magnification (Congo red staining). B, C – Aβ immunohistochemistry: the plaques in the temporal cortex, nuclei are stained by hematoxylin. D - Tau immunohistochemistry: neurofibrillary tangles in the temporal cortex and at higher magnification, nuclei are stained by hematoxylin.

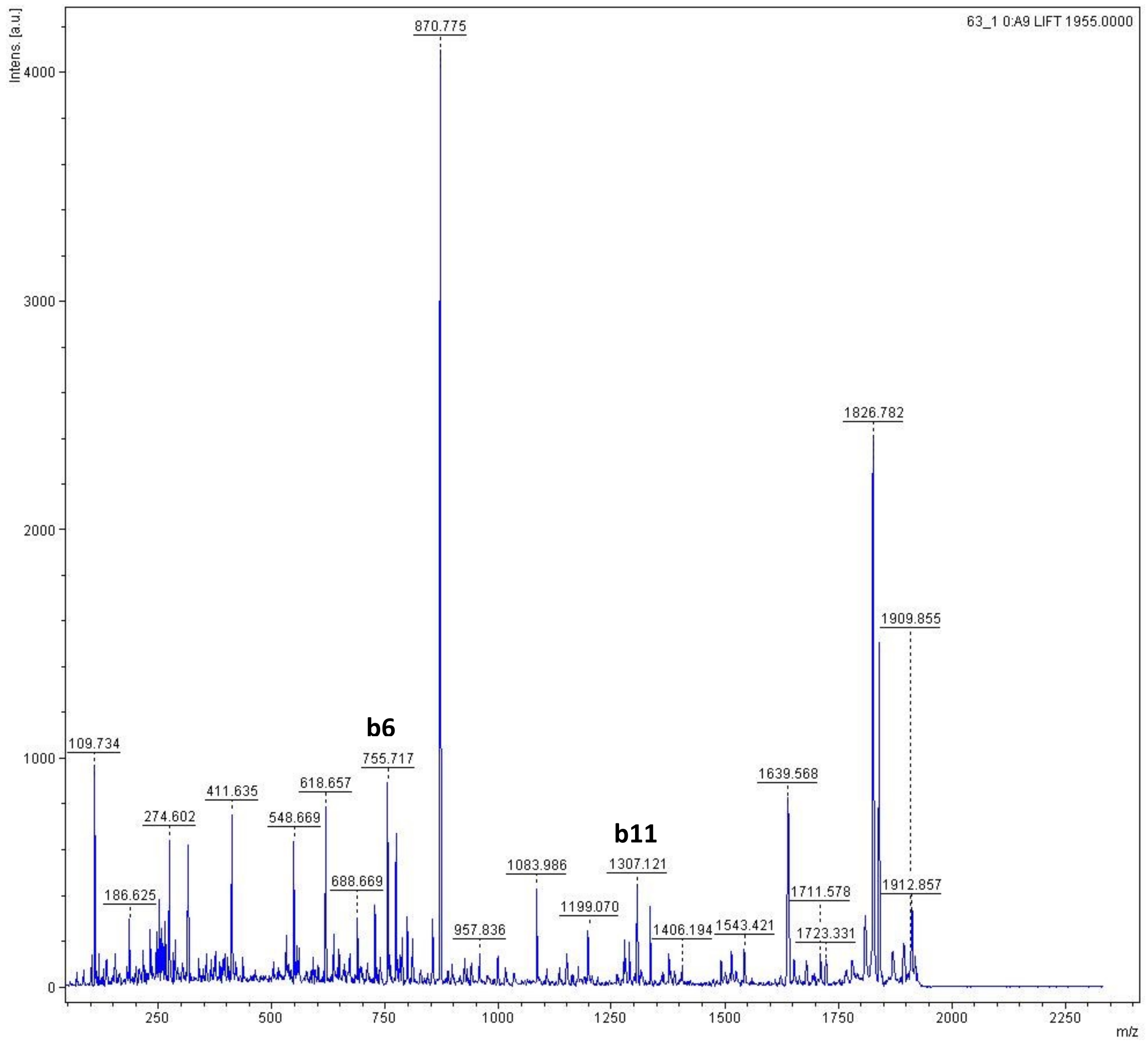

Figure S10. MALDI TOF/TOF mass-spectrum of Aβ (1-16) peptide from murine brain. The fragments used in further analysis are marked

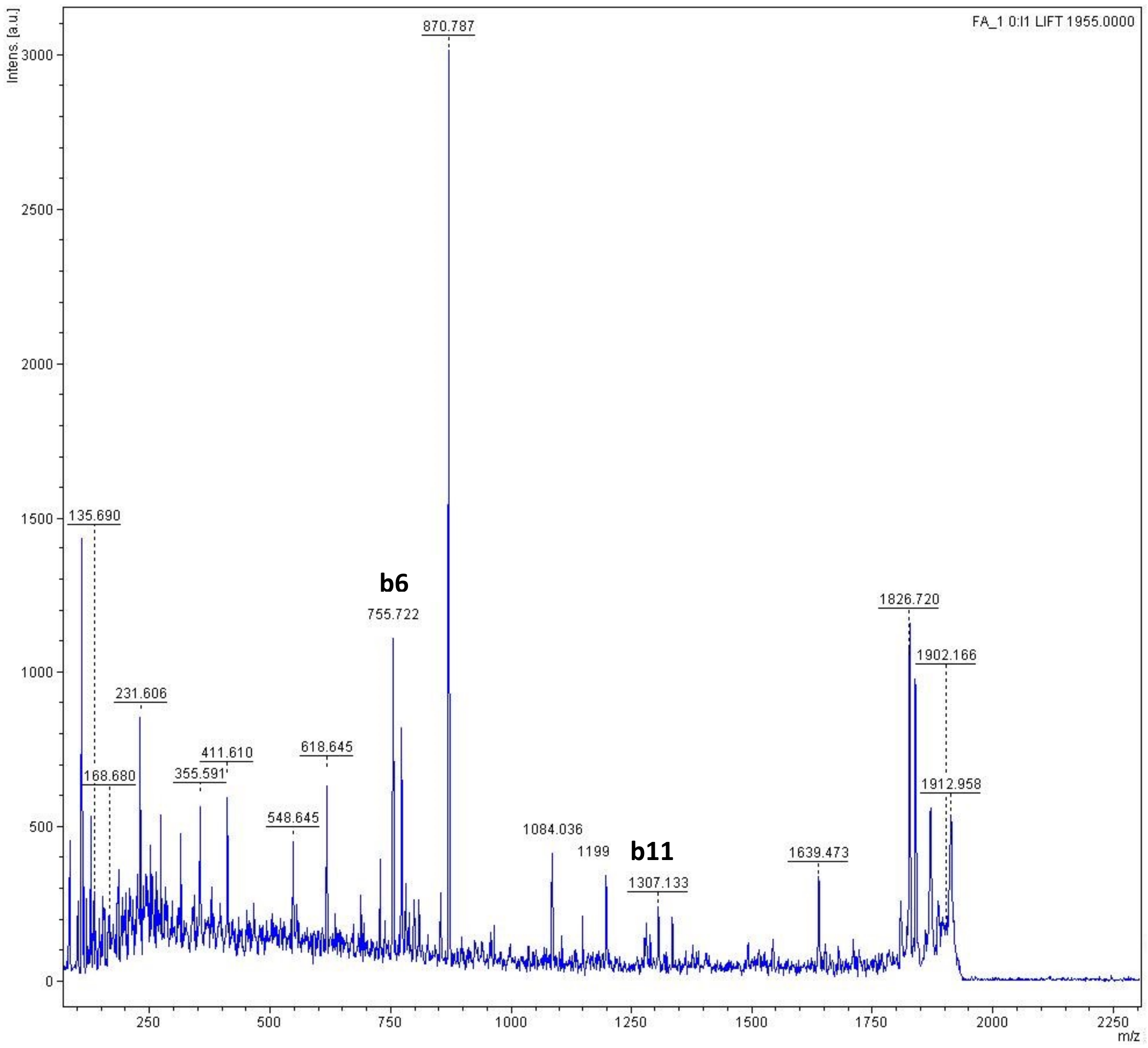

Figure S11. MALDI TOF/TOF mass-spectrum of Aβ (1-16) peptide from human brain. The fragments used in further analysis are marked.

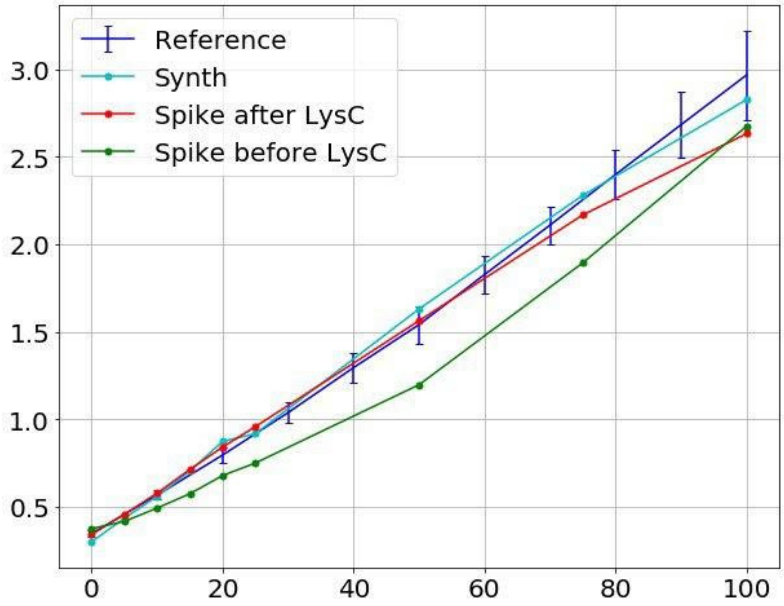

Figure S12. Comparison of calibration curves built from MALDI-TOF MS/MS data for Aβ (1- 16).

The dark blue line corresponds to “original” curve from previously reported study (Ivanov) built for binary mixtures of synthetic isoAsp7- Aβ (1- 16)/norm Aβ (1- 16) using b6/b11 fragments to estimate iso/norm Aβ ratio, light blue – analogous curve built for mixtures of synthetic Aβ standards during current study, green – for binary iso/norm Aβ (1-42) mixtures spiked before LysC hydrolysis into SPE eluates of wild type murine brain samples (resulted mixtures contain Aβ (1-16)), red –for for binary iso/norm Aβ (1-16) mixtures spiked after LysC hydrolysis into wild type brain SPE eluate.

Table S1. MALDI TOF/TOF data for isoD7/norm Aβ ratio evaluation.

5xFAD age (months)	Mean Area(b6)/Area(b11)	Std	Mean isoD7 Aβ(%)	Median isoD7 Aβ(%)	Std (%)
4	0.541626	0.026086	8.445072	8.357990	1.033886
7	0.536257	0.026647	8.211592	8.203509	1.131656
7	0.549125	0.025667	8.769811	8.540421	1.084700
7	0.553862	0.082327	8.948203	8.172308	3.454167
8	0.614373	0.062268	11.562013	10.886437	2.578358
9	0.564969	0.038225	9.451288	9.563768	1.606382
9	0.612002	0.068110	10.859800	10.343871	3.057531
10	0.627873	0.059568	12.139163	11.527617	2.464371
10	0.649018	0.069793	13.032130	12.939922	2.873765
11	0.691812	0.049218	14.848794	14.374113	2.002937
11	0.655065	0.024810	13.306304	13.413654	1.025988
12	0.710946	0.065573	15.643281	15.685687	2.679482
12	0.675253	0.052513	14.150281	14.295534	2.160335
14	0.663384	0.058474	13.646403	13.071600	2.408961
14	0.684669	0.059977	14.543407	14.890721	2.466280
16	0.726371	0.059809	16.290842	16.554508	2.438911
16	0.720023	0.079984	16.014123	16.054041	3.262080
18	0.97807349	0.04733925	26.528029	26.859970	1.824111
23	1.08881689	0.06249227	30.859565	30.660435	2.343312
human	2.407290	0.283563	75.753073	76.153331	8.418399
